# Assessment and forecasting of water ecological security and obstacle factor diagnosis in the Hexi Corridor of Northwest China

**DOI:** 10.1038/s41598-024-74925-0

**Published:** 2024-10-09

**Authors:** Dongyuan Sun, Zonghu Ji, Yike Wang, Wenrui Zhang

**Affiliations:** College of Water Conservancy and Hydropower Engineering, Gansu Agricultural University, No. 1 Yingmen Village, Anning district, Lanzhou, Gansu 730070 China

**Keywords:** Pressure-state-response model, Water ecological security, Comprehensive index, Obstacle factors, Grey model, Environmental social sciences, Hydrology

## Abstract

The water ecological security pattern is a core factor. A scientific, accurate, and practical evaluation of water ecological security provides a theoretical basis for regional water ecological management. Using water resource data from five cities in the Hexi Corridor of Gansu Province (Jiuquan (JQ), Jiayuguan (JYG), Zhangye (ZY), Jinchang (JC), and Wuwei (WW)) from 2006 to 2021, a water ecological security evaluation index system based on the PSR (pressure-state-response) framework was constructed, covering 27 factors related to water resources, socio-economics, and the ecological environment. The main obstacle factors of water ecological security were identified using the obstacle degree model, and the grey GM(1,1) model was employed to predict water ecological security. Results indicated that the comprehensive assessment index of water ecology in the Hexi Corridor increased from 2006 to 2021, showing a transition from relatively unsafe (0.319) to basic safety and then to relatively safe (0.672). The pressure and response systems were the main limiting factors affecting water ecological security in the Hexi Corridor. After a slight decline in 2008, the overall spatial distribution continued to rise, with WW City and ZY City leading since 2016. ZY had a higher safety grade proportion (25%) compared to other areas in the Hexi region. The pressure system was the most significant obstacle to water ecological security after 2006. Prediction results indicated that the comprehensive evaluation index of water ecological security would continue to rise annually from 2022 to 2031, reaching a very safe level by 2025. The evaluation results provide a scientific basis for ecological security and risk decision-making in the study area.

## Introduction

As a complex and highly interconnected natural-social-economic system, the water ecosystem links the earth’s soil, lithosphere, atmosphere, and biosphere. As one of the most biologically active and diverse habitats, water ecological security underpins regional ecological security and is crucial for maintaining the stability of ecological factors and promoting sustainable man-land system development^1–4^. Assessing water ecological security is vital for this field and provides key insights for regional sustainable development. Currently, the negative effects of climate warming and worsening drought are becoming increasingly severe. The stable supply of water resources is shrinking, and the demand gap is widening due to population growth and accelerated urbanization^5,6^. With increasing national attention to ecological security, assessing water ecological security in Northwest China’s arid regions is crucial. This evaluation aims to provide countermeasures to promote healthy and sustainable water ecosystem development and address the supply-demand imbalance of water resources in arid areas.

Water ecological security is a global concern, closely linked to regional ecosystem stability, economic development, and human health. Many scholars have conducted extensive research on water ecological security from various perspectives and using different methods. Currently, typical evaluation systems include the Analytic Hierarchy Process (AHP), index species ecosystem evaluation, the Pressure-State-Response (PSR) model, and fuzzy comprehensive evaluation^7^. The AHP was used to systematically evaluate water ecological security in the upper, middle, and lower reaches of the Xiangjiang River Basin, providing a scientific basis for water ecological protection^8^. Ecological quality was assessed using a multi-scale index method based on ecological indicators^9^. The Relative Risk Model (RRM) was used to analyze ecological risks in the water environment^10^. A comprehensive assessment of water quality and the ecosystem was conducted by integrating physicochemical and hydromorphological data^11^. Ecological security in the Pearl River Delta region from 1990 to 2015 was evaluated using the PSR model^12^. Seventeen cities in Shandong Province were studied by constructing a water ecological security index system and using a fuzzy comprehensive evaluation method to assess their water ecological security. However, existing studies rarely identify obstacle factors or conduct predictive analysis; they primarily focus on establishing evaluation index systems to assess water ecological security levels. In response, this study constructs an obstacle factor identification model for the northwest inland river region to analyze key factors affecting water ecological security and development, enhancing the systematic understanding of water ecological security in the study area. This approach improves the theoretical and practical application value of the evaluation system.

In arid inland river basins, research is limited due to unique geographical conditions, fragile ecosystems, and water scarcity; this is especially true for evaluating and predicting water ecological security in the Hexi Corridor. The Hexi Corridor, a typical northwest inland arid area, serves as a critical ecological security barrier and is vital for the Belt and Road Initiative^13^. Despite its ecological advantages, the region faces challenges due to high population density, intensive water use, supply-demand conflicts, and significant ecological issues, leading to a decline in hydrological and ecological functions^14^. The trend of “partial improvement but overall degradation” in ecosystem functions continues, with the ecological environment deteriorating and a risk of a systemic water crisis emerging. Thus, it is urgent to clarify the water ecological security status of the Hexi Corridor, identify key influencing factors, systematically evaluate its current state, and predict future trends to provide technical support for understanding regional water ecological security under changing conditions^15,16^.

This study provides valuable theoretical insights into water ecological security in the Hexi Corridor, highlighting global scholarly concern for sustainable development in the region. The study aims to (1) analyze the current situation and key influencing factors of water ecological security in the Hexi Corridor; (2) reveal the temporal and spatial characteristics of water ecological security; and (3) identify the dynamic change trends in water ecological security. Forecasting research can identify potential water ecological security risks in advance, providing a scientific basis for proactive management strategies and planning. The study serves as a case for identifying driving factors of inland water ecological changes in arid areas, offering a decision-making reference for ensuring water ecological security and optimal water resource allocation.

## Materials and methods

### Study area

**Figure 1:**
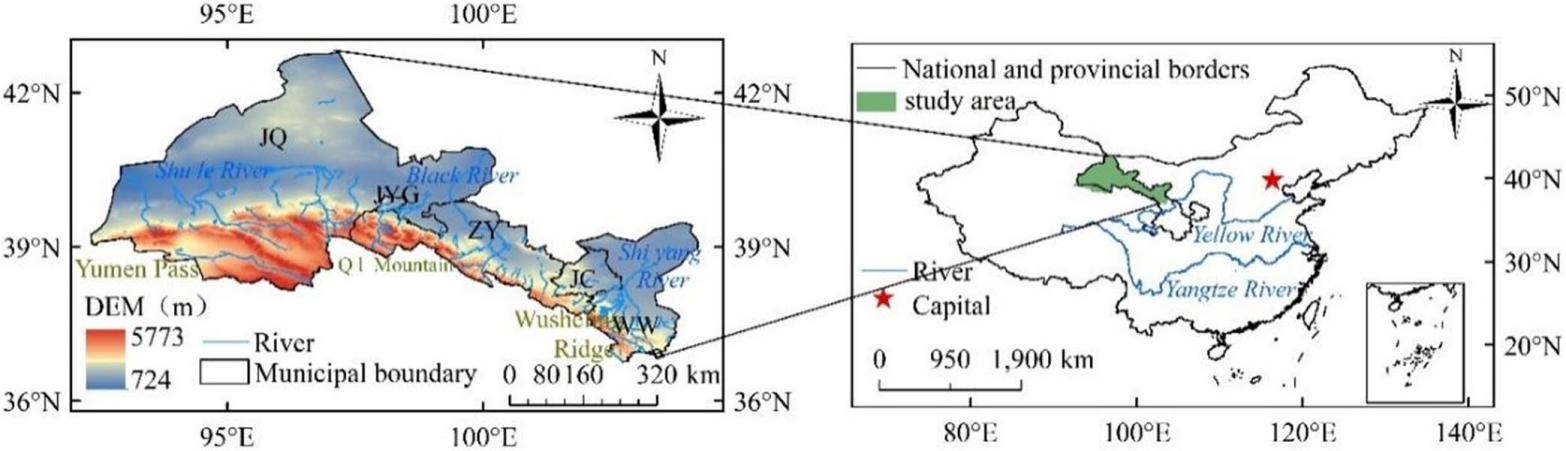
Overview of the Basin. Note: 1 The figure made in ARCGIS10.7 (https://desktop.arcgis.com/zh-cn/index.html), the using of natural resources reproduction standard mapping (http://211.159.153.75/index.html). The approval number is GS (2019) 3333, and the boundary of the base map is not modified.

The Hexi Corridor (37° 15’-41° 30’N, 92° 21’ -104° 45’E) is located between the Qilian Mountains and the Beishan Mountains in the northwest of Gansu Province, China (Fig. 1). It starts from Wushaoling in the east and ends at Yumenguan in the west. It is bounded by Qilian Mountains in the south and borders Inner Mongolia in the north. Geographically, from west to east are Wuwei, Jinchang, Zhangye, Jiayuguan, and Jiuquan. The terrain is high in the west and low in the east, the surrounding glacier resources, groundwater, and precipitation jointly breed the three major inland rivers of Shiyang River, Hei River, and Shule River, which pour into the corridor plain from west to east to form oases of varying sizes. Its altitude is 1139–3100 m, annual precipitation is 40–410 mm, and annual evaporation is 1500–3311 mm. The climate is dry with little rain and frequent sandstorms due to strong winds and sandstorms^17,18^.

### Data sources

The data mainly came from the “Gansu Statistical Yearbook”, “Gansu Water Resources Bulletin”, “Gansu Development Yearbook”, etc. from 2006 to 2021, among which the Gansu Statistical Yearbook and Gansu Development Yearbook are from the Gansu Provincial Bureau of Statistics (https://tjj.gansu.gov.cn/tjj/c109464/info_disp.shtml), and the water resources bulletin (https://slt.gansu.gov.cn/slt/c106726/c106732/c106773/c106775/tld.shtml?fLEvYk0FzGXg=1727268237052) is from the Gansu Provincial Water Resources Department.

### Methods

#### Building an indicator system and standardization

**Figure 2:**
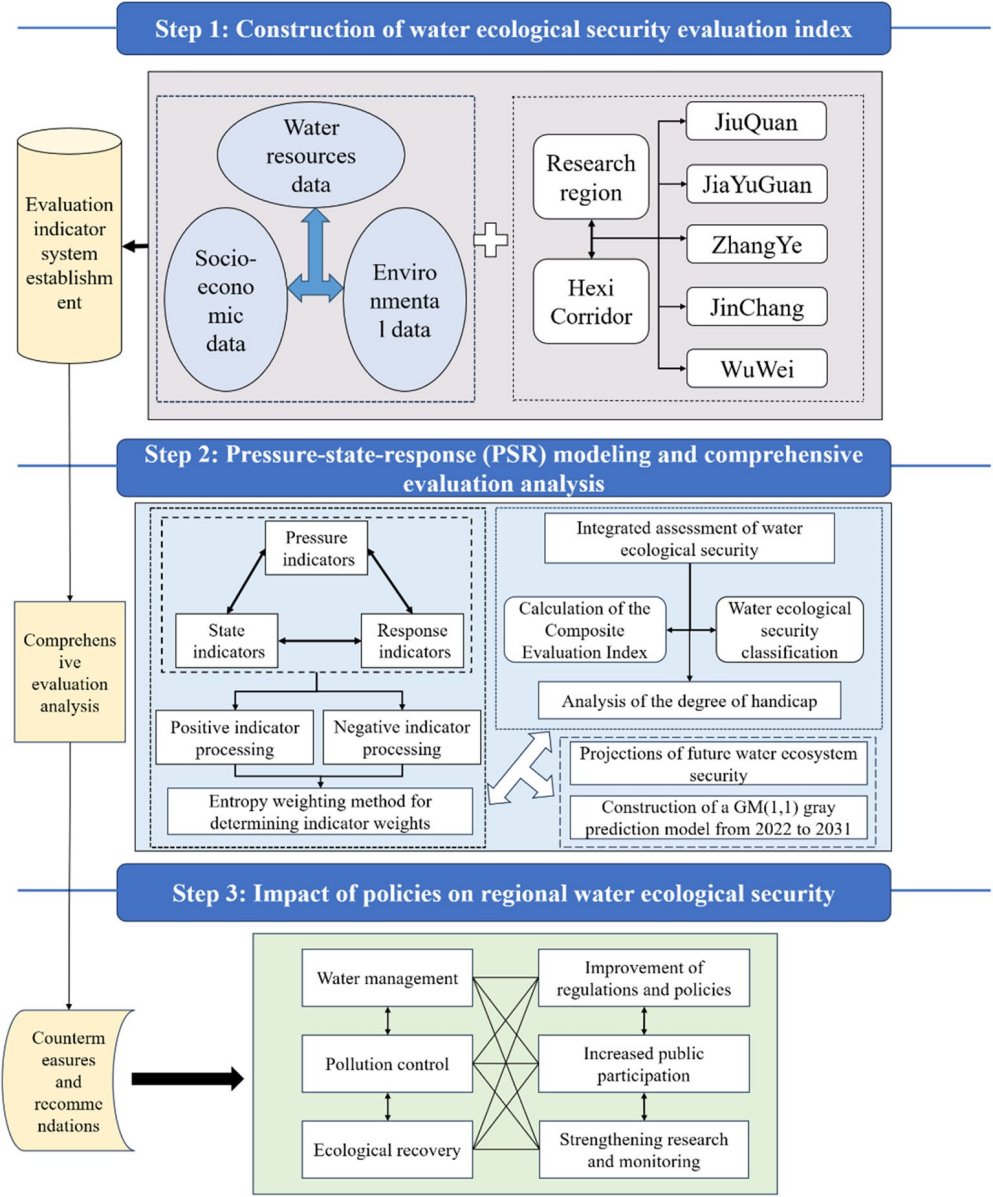
Flow chart of the established framework.

Based on the current water ecology status and prominent ecological problems in the Hexi Corridor, a three-tier water ecological security evaluation index system was proposed. The PSR (Pressure-State-Response) analysis model was applied, assigning causal logical relationships to indicators to enhance the theoretical and practical value of the evaluation system^19^. The Pressure (P) index represents factors where natural or human activities exert pressure on ecosystems and social systems, reflecting the impact of these pressures. The State (S) index indicates the current condition of society and ecosystems, representing the system’s health. The Response (R) index includes countermeasures taken when the system faces risk pressures. This framework illustrates the causal relationships in the socio-ecological system: pressures from natural and human factors alter the state of social-ecological systems, prompting responses through adaptive, preventive, and mitigative measures to sustain system stability. Pressure, state, and response are interconnected, reflecting the dynamic interactions between humans and the environment. Based on principles of purpose, comprehensibility, feasibility, stability, coordination, integration, scientific validity, comparability, sensitivity, and operability, and guided by the framework flow chart (Fig. 2), 27 indicators from the three PSR levels were selected to objectively assess the water ecological security of the Hexi Corridor (Table 1).

To facilitate calculations, the dispersion degree of each indicator was determined using entropy values^20^. The dispersion degree of each indicator was directly proportional to its impact (weight) on the comprehensive evaluation, and normalization was applied.

1 $$\:\mathrm{Positive}\;\mathrm{indicators}:\;X_{ij}^{'\:}=\frac{X_{ij}-min\left(X_{ij}\right)}{max\left(X_{ij}\right)-min\left(X_{ij}\right)}$$

2 $$\mathrm{Negative}\;\mathrm{indicator}:\:X_{ij}^{'\:}=\frac{max\left(X_{ij}\right)-X_{ij}}{max\left(X_{ij}\right)-min\left(X_{ij}\right)}$$

In the formula: $\:{X}_{ij}\:$ is the original indicator data, $\:{X}_{ij}^{{\prime\:}}\:$ is the standardized data, where i=(1, 2,… n); j=(1, 2,… m).

**Table 1:** Water ecological security evaluation index system of Hexi Corridor in Gansu Province

Target layer	Criterion layer	Indicator layer	Index Properties	Indicator Definition
Evaluation index system for water ecological security	Pressure	population(B1)	–	Reflect the impact of population pressure on water resources
		Water consumption for farmland irrigation(B2)	–	Reflect the agricultural water consumption situation in the region
		Water consumption of forestry, animal husbandry, fishing, and livestock(B3)	–	Reflect the water consumption status of forestry, animal husbandry, fishing, and livestock in the region
		Industrial Water(B4)	–	Reflect the industrial water consumption situation in the region
		Residential water consumption(B5)	–	Reflect the water use situation of the population in the region
		eco-environmental water use(B6)	+	Reflect the water consumption status of the ecological environment in the region
		total water consumption(B7)	–	Reflect the dynamic changes in regional water use
		Annual sewage discharge(B8)	–	Reflect the situation of regional sewage treatment
		Urban public water use(B9)	–	Reflecting urban water supply capacity
		Urban environmental water consumption(B10)	+	Reflect the water consumption situation of regional urban environment
	State	Per capita water consumption(B11)	–	Total amount of domestic water used by residents/total populationReflect the degree of water security for residents
		Groundwater resources(B12)	+	Reflect the amount of groundwater resources in the region
		Water Resources(B13)	+	Reflect the total amount of water resources in the region
		Surface water resources(B14)	+	Reflect the amount of surface water resources in the region
		annual precipitation(B15)	+	Reflect the basic precipitation situation in the region
		Actual irrigation area of farmland(B16)	+	Reflect the actual agricultural water consumption situation in the region
		GDP(B17)	+	Reflect the situation of regional construction capacity
		per capita GDP(B18)	+	Reflecting the overall economic development of the region
		Industrial added value(B19)	+	Reflect the industrial development situation in the region
	Response	Ratio of tertiary industry to GDP(B20)	+	Reflect the proportion of the tertiary industry in the total output value
		total grain output(B21)	+	Reflecting the constraints of water resources on food production
		Urbanization Rate(B22)		Reflecting the level of social development
		Water production coefficient(B23)	+	Reflect the precipitation conversion rate per unit area of the region
		Water production modulus(B24)	+	Total regional water resources/total regional area,Reflect the amount of water resources per unit area in a region
		Water-saving irrigation area(B25)	+	Reflect the development status of water-saving agriculture
		Water consumption per 10000 yuan of GDP(B26)	–	Reflecting the degree to which economic development utilizes water resources
		Water consumption for industrial added value of 10000 yuan(B27)	–	Industrial water consumption/industrial added value per 10000 yuan, reflecting dynamic industrial water use benefits

Note: “+” represents a positive indicator; “–” represents a negative indicator.

#### Entropy method

Reflecting the importance of indicators by their respective weights^21^, This study used the entropy method to calculate indicator weights. The specific formula was as follows:

Calculate the proportion of i sample values under the j indicator

3 $$\:{F}_{ij}=\frac{{X}_{ij}^{{\prime\:}}}{\sum\:_{i=1}^{n}{X}_{ij}^{{\prime\:}}}$$

Find the entropy value of the j indicator

4 $$\:{e}_{j}=-k\sum\:_{j=1}^{m}{F}_{ij}\times\:ln{F}_{ij}$$

Where, 0≤$\:{e}_{j}$≤1, Ej Information entropy: $\:k=\frac{1}{\text{l}\text{n}\left(n\right)}$

Define the weight of the j indicator

5 $$\:{w}_{j}=\frac{1-{e}_{j}}{\sum\:_{j=1}^{m}{e}_{j}}$$

Where, $$\:{w}_{j}$$∈ [0, 1], just $$\:\sum\:_{j=1}^{m}{w}_{j}=1$$

#### Index calculation and grading

Calculate the target layer score, namely the Water Ecological Security Index (ESI), by weighted summation of the evaluation index of the other case layer. Use this index to evaluate the overall ecological security status of the watershed^2–22^.

6 $$\:\text{E}\text{S}\text{I}=\sum\:_{j=1}^{m}{X}_{ij}^{{\prime\:}}\times\:{w}_{j}$$

The classification standards for water ecological security levels refered to the research results of relevant scholars, the classification of water ecological security levels in the Hexi Corridor was shown in Table 2.

**Table 2:** Classification of Water Ecological Security levels.

Comprehensive index of water ecological security	Security status classification	Water ecological security status
≥ 0.81	1	Safe
0.8–0.6	2	Safer
0.6–0.4	3	Basic safety
0.4–0.2	4	Less secure
0.2–0	5	Very unsafe

#### Obstacle model

To further explore the main obstacle factors affecting water ecological security^23,24^, the obstacle degree value was calculated through factor contribution and indicator deviation, and the specific formula was as follows:

7 $$\:{I}_{ij}=1-{X}_{ij}^{{\prime\:}}$$

8 $$\:{B}_{j}={w}_{j}$$

9 $$\:{H}_{ij}=\frac{{B}_{j}\times\:{I}_{ij}}{\sum\:_{i=1}^{n}({B}_{j}\times\:{I}_{ij})}$$

10 $$\:{H}_{j}=\sum\:_{i=1}^{n}{H}_{ij}$$

In the equation: $\:{H}_{ij}$、$\:{H}_{j}\:$Respectively represent the obstacle degree values of the impact of each indicator layer and criterion layer on water ecological security.

#### Grey prediction model

The grey prediction GM (1,1) model was applied to generate and process raw data to find the patterns of system changes, establish corresponding differential equation models, and predict the future development trend of things. This model was suitable for complex relationships with multiple data indicators that constrain and influence each other^25–27^.

(1) Collect raw data to form a sequence X^(0)^, and perform a cumulative generation process on the original sequence to obtain a new sequence X^(1)^.

(2) The differential equation corresponding to the GM (1,1) model was

11 $$\:\frac{d{X}^{\left(1\right)}}{dt}+a{X}^{\left(1\right)}=u$$

In the formula, t is the value of the nth sequence; A is a fixed number of values; U is the development grey number.

(3) Using the Least Squares Method to Solve Parameter Vectors

12 $$\widehat{\mathrm a}=\left(B^TB\right)^{-1}B^TY=\begin{pmatrix}a\\u\end{pmatrix}$$

In the formula, B is the data matrix; Y is the data vector.

(4) Building predictive model expressions

13 $$\:\widehat{\mathrm g}^{\left(1\right)}\left(t\right)=\left[X^{(0)}\left(1\right)-\frac ua\right]e^{-a\left(t-1\right)}+\frac ua$$

Where, t =(2, 3, …, n)。

Final predicted value obtained

14 $$\:\widehat{\mathrm g}^{\left(0\right)}\left(t\right)=\widehat{\mathrm g}\left(1\right)\left(t\right)-\widehat{\mathrm g}\left(1\right)(t-1)$$

In the equation, $\:\widehat{\mathrm g}\left(1\right)\left(t\right)$ is the predicted cumulative value; $\:\widehat{\mathrm g}^{\left(0\right)}\left(t\right)$ is the t-th predicted value of the evaluation index.

(5) Conduct accuracy testing. As shown in Table 3. The established GM (1,1) model had objective deviations from the actual sequence, and the applicability and effectiveness of the newly established model were judged by calculating the deviation accuracy of the model.

① Posteriori difference test model accuracy

15 $$\:C=\frac{{S}_{2}}{{S}_{1}}$$

In the formula, C is the posterior error ratio; $\:{S}_{2}$ is the residual variance; 𝑆_1_ is the variance of 𝑆^(0)^. Given that 𝐶_0_ > 0 and C<𝐶_0_ holds, model (1) passes a posterior error test at the 𝐶_0_ level.

② Small error probability p-test

16 $$\:p=P\left(\left|\varepsilon\left(k\right)-\overline\varepsilon\right.\right)<0.6745S_1$$

Given 𝑃_0_ > 0, if p>𝑃_0_ holds, then model (1) passes the small error probability test with probability 𝑃_0_.

**Table 3:** GM (1,1) model accuracy inspection standards.

project	Model accuracy level			
	good	qualified	reluctantly	unqualified
C(Posterior error ratio)	<0.35	<0.50	<0.65	≥ 0.65
P(Small error probability)	>0.95	>0.80	>0.70	>0.60

## Results

### Weight of evaluation index

**Figure 3:**
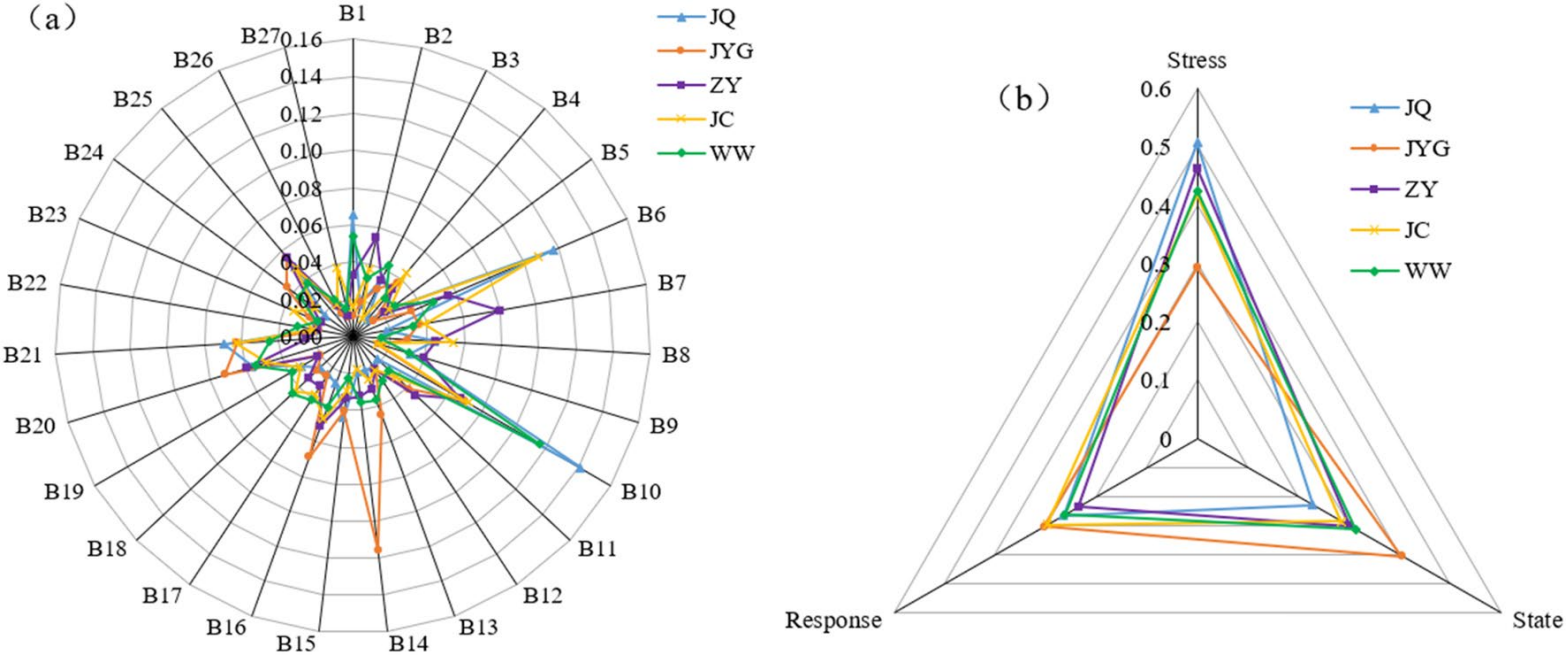
Indicator weight radar.

Table 4 and Fig. 3a present the three most impactful indicators on water ecological security for each city in the Hexi Corridor. For JQ City, the key indicators were ecological water use (0.1173), urban environmental water use (0.1410), and total grain production (0.0694), accounting for 11.7%, 14.1%, and 6.9%, respectively, with a cumulative weight of 32.7%. For ZY City, total water consumption (0.0180), urban environmental water consumption (0.0669), and the proportion of tertiary industry to GDP (0.0599) were key, accounting for 8.0%, 6.7%, and 6.0%, respectively, with a cumulative weight of 21.2%. JC City’s main indicators were ecological environment water use (0.1081), urban environmental water (0.0713), and total grain production (0.0629), accounting for 10.8%, 7.1%, and 6.3%, respectively, with a cumulative weight of 24.2%. WW City’s key factors were population (0.0536), urban environmental water consumption (0.1159), and the proportion of tertiary industry to GDP (0.0550), accounting for 5.4%, 11.6%, and 5.5%, respectively, with a cumulative weight of 22.5%. The least impactful indicators for JQ City were industrial water use, water use per 10,000 yuan of GDP, and water use per 10,000 yuan of industrial added value, with a cumulative weight of 4.0%. For JYG City, population, residential water consumption, and industrial value-added water consumption per 10,000 yuan had a cumulative weight of 3.5%. In ZY City, the water production coefficient, water consumption per 10,000 yuan of GDP, and industrial added value water consumption per 10,000 yuan contributed a cumulative weight of 4.8%. JC City’s least impactful indicators were population, water consumption for forestry, animal husbandry, fishing, and livestock, and urban public water use, with a cumulative weight of 4.1%. WW City’s least impactful indicators included annual sewage discharge, water production coefficient, and industrial value-added water consumption per 10,000 yuan, with a cumulative weight of 5.2%. According to Fig. 3b, the weights of the pressure systems in the five cities were JQ (50.7%), JYG (29.2%), ZY (46.3%), JC (41.9%), and WW (42.5%). The state system weights were JQ (22.8%), JYG (40.5%), ZY (30.1%), JC (28.4%), and WW (31.3%). The response system weights were JQ (26.5%), JYG (30.2%), ZY (23.5%), JC (29.7%), and WW (26.2%). The main driving systems for water ecological security in JQ City and JC City were the pressure and response systems. In ZY City and WW City, the pressure and state systems were the primary drivers of water ecological security. For JYG City, the state and response systems were the primary driving forces for water ecological security. In summary, water ecological security in the Hexi region was primarily influenced by ecological environment water use, urban environmental water use, surface water resources, the proportion of tertiary industry to GDP, and total grain production, highlighting the significant impact of human activities. The impact of subsystems on water ecological security in the Hexi Corridor followed the order: pressure system > state system > response system. Except for the JQ area, the impact of subsystems on water ecological security in the other four cities was relatively stable.

**Table 4:** Weights of water safety assessment indicators in the five cities of Hexi.

Index	JQ	JYG	ZY	JC	WW	Index	JQ	JYG	ZY	JC	WW
**B1**	0.0655	0.0107	0.0330	0.0157	0.0536	**B15**	0.0441	0.0406	0.0337	0.0294	0.0225
**B2**	0.0179	0.0191	0.0548	0.0374	0.0320	**B16**	0.0270	0.0689	0.0510	0.0471	0.0402
**B3**	0.0350	0.0286	0.0337	0.0110	0.0429	**B17**	0.0244	0.0254	0.0321	0.0370	0.0407
**B4**	0.0117	0.0376	0.0329	0.0442	0.0267	**B18**	0.0245	0.0267	0.0331	0.0426	0.0449
**B5**	0.0244	0.0139	0.0215	0.0242	0.0279	**B19**	0.0324	0.0201	0.0216	0.0325	0.0377
**B6**	0.1173	0.0343	0.0560	0.1081	0.0469	**B20**	0.0565	0.0718	0.0599	0.0487	0.0550
**B7**	0.0180	0.0360	0.0801	0.0391	0.0327	**B21**	0.0694	0.0624	0.0254	0.0629	0.0449
**B8**	0.0440	0.0292	0.0445	0.0538	0.0149	**B22**	0.0211	0.0188	0.0191	0.0217	0.0302
**B9**	0.0324	0.0143	0.0399	0.0136	0.0316	**B23**	0.0213	0.0252	0.0185	0.0344	0.0207
**B10**	0.1410	0.0690	0.0669	0.0713	0.1159	**B24**	0.0188	0.0441	0.0271	0.0250	0.0359
**B11**	0.0175	0.0413	0.0463	0.0320	0.0264	**B25**	0.0490	0.0555	0.0553	0.0478	0.0375
**B12**	0.0195	0.0215	0.0214	0.0215	0.0283	**B26**	0.0144	0.0141	0.0184	0.0190	0.0221
**B13**	0.0192	0.0447	0.0300	0.0249	0.0362	**B27**	0.0145	0.0103	0.0116	0.0376	0.0158
**B14**	0.0193	0.1157	0.0322	0.0174	0.0357						

Note: JQ represents Jiuquan, JYG represents Jiayuguan, ZY represents Zhangye, JC represents Jinchang, and WW represents Wuwei.

### Analysis of PSR model subsystem evaluation index

**Figure 4:**
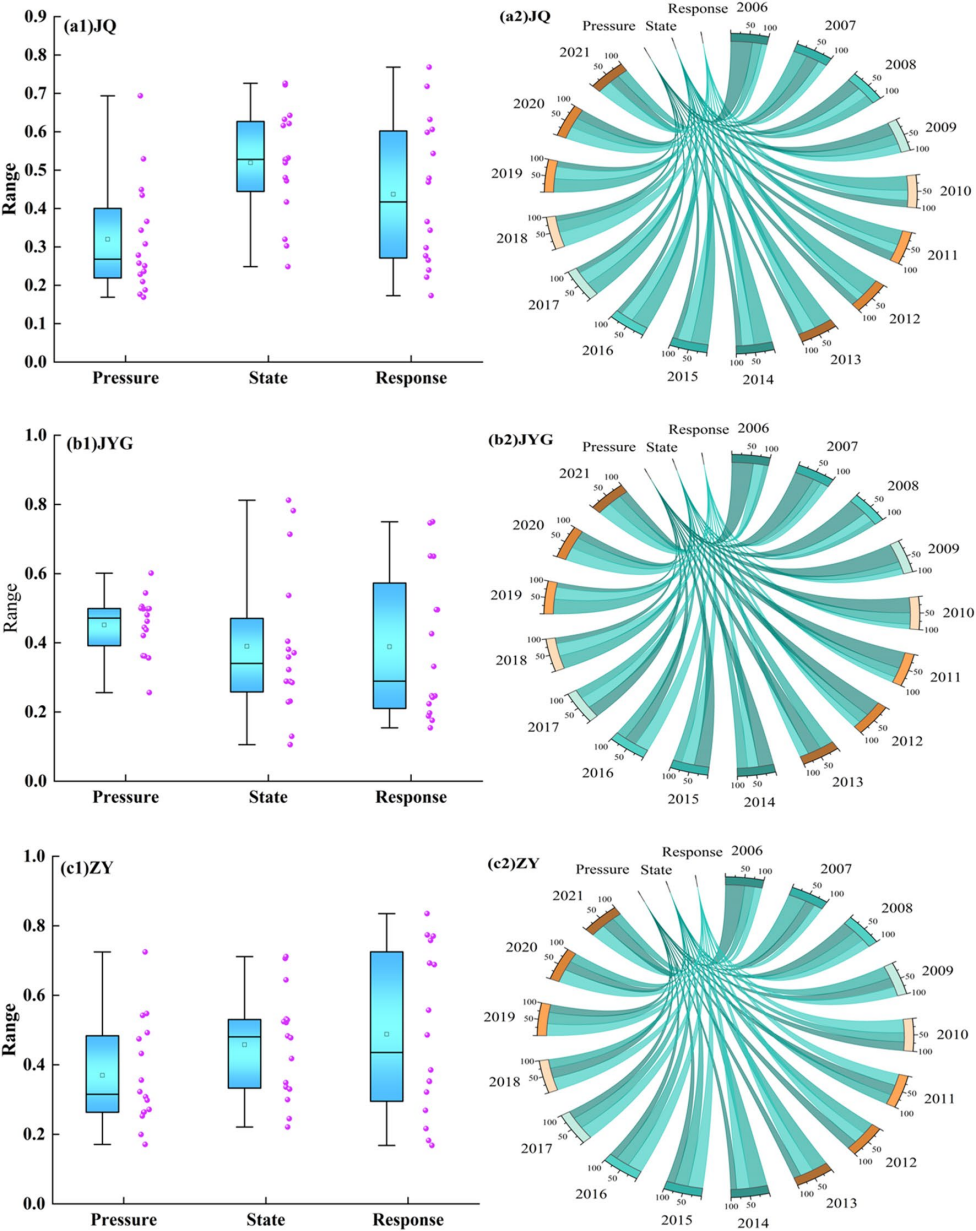

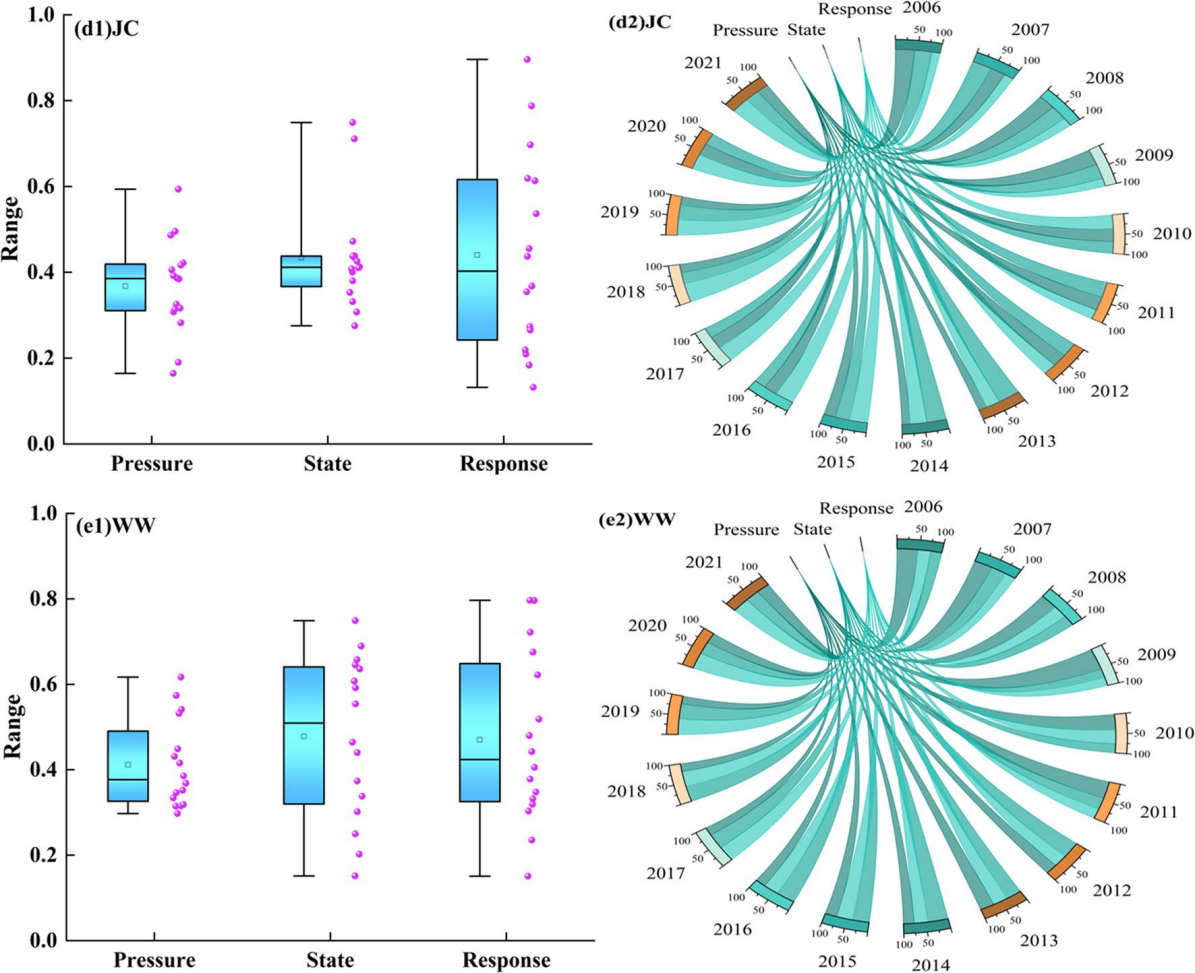
Trend of comprehensive index changes in the PSR system.

The state and response system curves in JQ City, JYG City, and WW City exhibited a fluctuating upward trend (Fig. 4). The pressure system in JQ City decreased from 0.694 in 2006 to 0.529 in 2021, reaching a minimum of 0.169 in 2014. This suggests that human activity pressure significantly impacts water ecological security. Since 2010, the pressure system’s proportion in the water ecological security index has been the smallest, and by 2020, the gap among the three systems narrowed continuously. The pressure system in JYG City showed a downward trend during 2009–2013 and 2017–2019. The fluctuation increased due to rising population (B1), water consumption for forestry, animal husbandry, fishing, and livestock (B3), total water consumption (B7), annual sewage discharge (B8), and decreased urban environmental water consumption (B10). From 2006 to 2012, the pressure system had the highest proportion in the water ecological security index, declining to 24.76% by 2021. In WW City, the minimum values of the state and response systems appeared simultaneously in 2009, mainly due to significant changes in groundwater resources (B12), total water resources (B13), GDP (B17), the proportion of tertiary industry to GDP (B20), and total grain production (B21). From 2006 to 2011, the pressure system had the highest proportion in the water ecological security index, after which it alternated, and the gap continued to narrow. The proportions of the comprehensive index of the three cities and three major systems gradually became equal.

The comprehensive index of the three major systems in ZY City and JC City fluctuated significantly, with the state system in ZY City showing marked fluctuations in 2007 and 2017–2020. These fluctuations were driven by significant changes in per capita water consumption (B11), annual precipitation (B15), actual farmland irrigation area (B16), total water resources (B13), and surface water resources (B14), causing the maximum proportions of each system in the water ecological security index to alternate. In JC City, the response system’s index increased sharply from 2013 to 2019, driven by annual increases in total grain output (B21), urbanization rate (B22), water production coefficient (B23), and water-saving irrigation area (B25). The proportion of the pressure system in the water ecological security index decreased, while the response system’s proportion increased. In 2021, the pressure system in JC City accounted for 27%, while the state system in ZY City accounted for 25.33%.

This indicates that human activities have a dominant impact on water ecological security across the Hexi region, with JQ and JYG particularly affected by the pressure system. This aligns with the increasing fragility of water ecological security as the region extends northwestward.

### The spatiotemporal variation trend of the comprehensive index of water ecological security

#### The temporal trend of the comprehensive index of water ecological security

**Figure 5:**
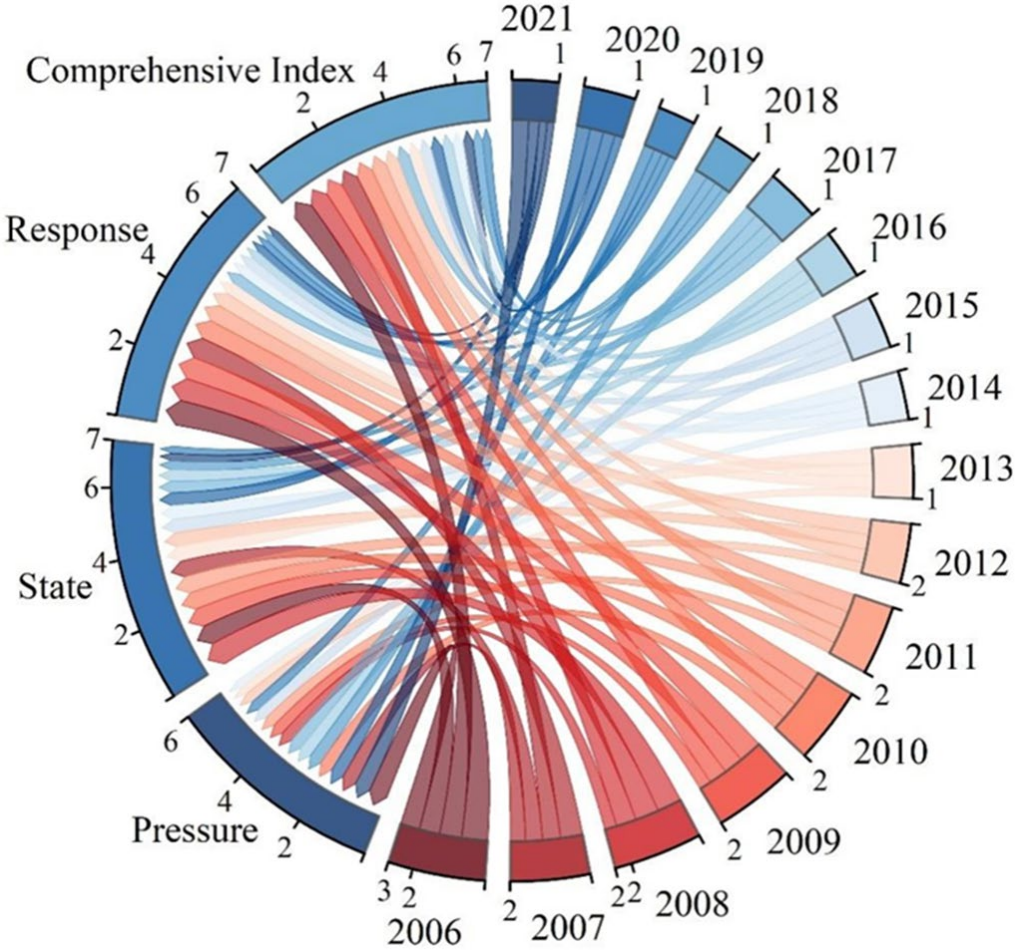
Trend of comprehensive index changes in the PSR system.

The water ecological security status of the Hexi Corridor has fluctuated from less secure to moderately safe and then to more secure over the past 16 years (Fig. 5). The overall comprehensive evaluation index of water ecological security has shown a continuous upward trend, indicating significant improvement. The comprehensive index increased from 0.3186 in 2006 to 0.6178 in 2021, with the highest value of 0.671 in 2021 and the lowest of 0.2599 in 2008, mainly influenced by fluctuations in the pressure and state systems. Between 2020 and 2021, the pressure system index increased by 28.2%, outpacing the state system (21.9%) and response system (18.7%). Conversely, between 2007 and 2008, the state system index decreased by 45.1%, exceeding the declines in the pressure (21.3%) and response systems (11.0%).The pressure system displayed a downward trend from 0.5011 in 2006 to 0.2615 in 2013. During this period, total water consumption, annual sewage discharge, and ecological water use increased, resulting in a lower pressure system score, which began to recover after 2014. Since 2015, the pressure and response systems have been the primary factors influencing water ecological security. The evaluation curve closely aligns with the state and response trends, indicating that water ecological security in the Hexi region has been significantly driven by the state system.

**Figure 6:**
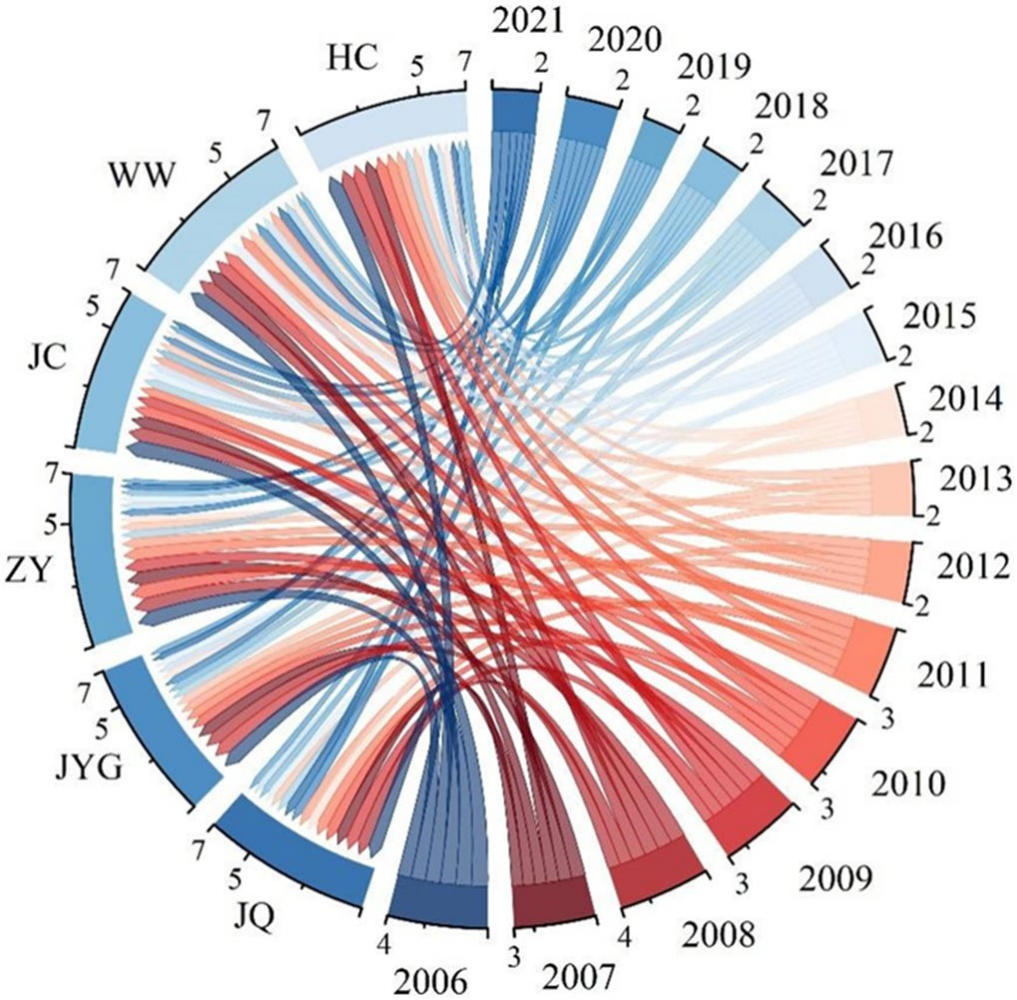
Trends in the comprehensive index of water ecological security in the Hexi Corridor.

The average comprehensive evaluation indices of the five cities in Hexi are as follows: WW (0.4535) > ZY (0.4356) > Hexi region (0.4283) > JQ (0.4254) > JC (0.4141) > ZY (0.4101), indicating a basic level of safety (Fig. 6; Table 5). From 2006 to 2021, except for JQ, the comprehensive evaluation indices of water ecological security for the other four cities ranged between 2 and 8. JQ’s indices ranged between 2 and 6, with peak values in 2021. In most other years, the indices generally showed an upward trend. Notably, the declines from 2007 to 2008 and 2019 to 2020 were primarily due to decreases in the state system, influenced by factors such as groundwater resources (B12), total water resources (B13), surface water resources (B14), and annual precipitation (B15). The unsafe distribution rates for Hexi region, JQ, JYG, ZY, JC, and WW were 56.25%, 43.75%, 56.25%, 56.25%, 56.25%, and 25%, respectively. The rates of basic safety were 31.25%, 56.25%, 31.25%, 18.75%, 31.25%, and 62.5%, while the relatively safe proportions were 12.5%, 0%, 12.5%, 25%, 12.5%, and 12.5%. Over time, the entire region and its cities have moved towards a safer state of water ecological security. This positive trend is likely due to the implementation of strict water management systems, river and lake management, enhanced pollution control, and ecological restoration efforts.

**Table 5:** Comprehensive Evaluation Index of Water Safety in the five cities of Hexi.

Year	JiuQuan	JiaYuGuan	ZhangYe	JinChang	WuWei	Hexi Corridor
2006	0.3897	0.2833	0.2738	0.2383	0.4077	0.3186
2007	0.4013	0.3329	0.3208	0.3244	0.4250	0.3609
2008	0.2858	0.2737	0.2267	0.2683	0.2450	0.2599
2009	0.3208	0.3394	0.2962	0.3469	0.2236	0.3054
2010	0.3835	0.3467	0.3815	0.4077	0.3531	0.3745
2011	0.2975	0.3207	0.3202	0.3640	0.4112	0.3427
2012	0.3208	0.3066	0.3052	0.3890	0.5105	0.3664
2013	0.4326	0.3252	0.3232	0.2808	0.3281	0.3380
2014	0.3437	0.3721	0.3592	0.3318	0.4376	0.3689
2015	0.4241	0.4045	0.4599	0.3636	0.4083	0.4121
2016	0.4979	0.4250	0.5629	0.4200	0.5033	0.4818
2017	0.5054	0.4666	0.6503	0.5025	0.5212	0.5292
2018	0.5467	0.6293	0.5725	0.4746	0.5721	0.5590
2019	0.5553	0.5727	0.6539	0.6512	0.6435	0.6153
2020	0.5093	0.5164	0.6158	0.5287	0.5749	0.5490
2021	0.5920	0.6468	0.6962	0.7335	0.6905	0.6718
Average	0.4254	0.4101	0.4386	0.4141	0.4535	0.4283

#### The spatial variation trend of the comprehensive index of water ecological security

**Figure 7:**
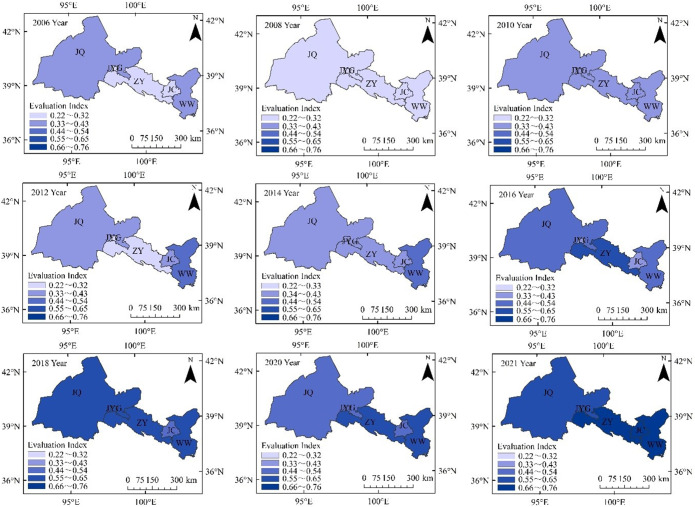
Spatial distribution of comprehensive development index. Same as Fig. 1.

The comprehensive water ecological indices for various cities in 2006, 2007, 2009, 2011, 2013, 2015, 2017, 2019, and 2021 were analyzed for spatial processing, as shown in Fig. 7. From 2006 to 2021, water ecological security in the Hexi Corridor exhibited a fluctuating upward trend, with a notable increase from 2014 to 2021. The years 2006–2012 saw relatively moderate fluctuations, with the worst security in 2008. WW City consistently maintained a leading position in water ecological security. In 2006, its comprehensive index was relatively low, and both WW and JQ were at the highest level of relative insecurity. At that time, the water ecological security status across the Hexi region was relatively unsafe. By 2021, JQ’s ecological safety level had improved to basic safety, while other cities and regions achieved a relatively safe level. The water ecological security indices for JQ, JYG, ZY, JC, and WW fluctuated between 0.2858 and 0.5920, 0.2737–0.6468, 0.2267–0.6962, 0.2383–0.7335, and 0.2236–0.6905, respectively. WW and JC cities exhibited a steady increase in their indices, with a minor decrease in 2008. Conversely, JQ and JYG experienced downward trends in 2008 and 2020, showing overall fluctuations and increases, indicating relative instability in local water ecological security. Since 2016, ZY and WW have maintained a leading position in water ecological security, with overall safety conditions being somewhat better in the eastern cities compared to the western ones.

Overall, water ecological security in the Hexi Corridor has improved over time. By 2021, JQ, WW, and ZY cities had better water ecological security than JC and JYG, evolving from relatively unsafe levels in 2006 to relatively safe levels. Despite fluctuations in the water ecological security index of some cities, the overall 16-year development shows that the ecological environment, water resources, and socio-economic relationships have gradually harmonized, with improved interactions. Additionally, the focus of the comprehensive water ecological security index has shifted eastward. Significant differences in the ecological security trends among cities in the Hexi Corridor should be addressed individually. This indicates that there is still room for improvement in the region’s ecological security. National and regional governments need to implement effective water ecological protection policies and measures to ensure sustainable development and establish a secure water ecological pattern along the “Silk Road Economic Belt.”

### Analysis of changes in Water Ecological Security Index and obstacle factors

**Figure 8:**
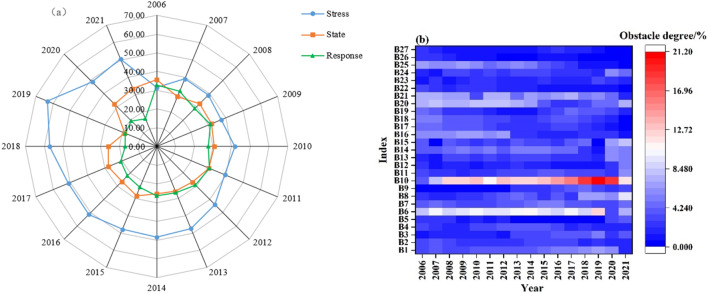
Changes and analysis of obstacle factors in water ecological security.

Except for 2006, the pressure system consistently had the highest obstacle degree and played a dominant role in water ecological security (Fig. 8a; Table 6). The primary obstacle factors affecting water ecological security were ecological water use (B6) and urban environmental water use (B10). In 2006, the state system had the highest obstacle degree, primarily due to the actual irrigation area of farmland (B16) and per capita GDP (B18). The obstacle degree of the pressure system in the Hexi region has been increasing annually, reaching 50.53% by 2021 with a 1.2% growth rate, making it the primary factor restricting local water ecological security. In contrast, the obstacle degree of the state and response systems has been decreasing annually, with the state system remaining relatively stable. The highest obstacle degrees for the state and response systems occurred in 2006, at 35.69% and 32.88%, respectively. Future efforts should focus on the pressure subsystem to further enhance water ecological security.

**Table 6:** Obstacle factors for water ecological security in the Hexi Corridor.

Year	Pressure system	State system	Response system
2006	31.44	35.69	32.88
2007	39.11	28.80	32.09
2008	38.83	32.42	28.75
2009	37.26	31.77	30.97
2010	41.84	30.60	27.56
2011	39.38	30.23	30.38
2012	44.02	26.95	29.03
2013	47.46	25.87	26.67
2014	48.34	25.16	26.50
2015	47.95	28.46	23.59
2016	51.42	26.44	22.14
2017	51.08	28.17	20.75
2018	57.19	25.86	16.95
2019	63.23	18.60	18.17
2020	48.70	32.03	19.27
2021	50.53	33.47	16.00

The results of the obstacle degree model analysis of driving factors affecting water ecological security in the Hexi Corridor are presented in Fig. 8b; Table 7. Table 7 shows that the top four obstacle factors are primarily from the pressure and response subsystems. Key obstacle factors for water ecological security in the Hexi region in 2006 and 2021 included ecological water use (B6), urban environmental water use (B10), proportion of tertiary industry to GDP (B20), total grain output (B21), actual irrigation area of farmland (B16), water-saving irrigation area (B25), population (B1), and annual sewage discharge (B8). Figure 7b indicates that annual sewage discharge (B8), urban environmental water consumption (B10), and annual precipitation (B15) increasingly impacted water ecological security. The impacts of actual irrigation area of farmland (B16), proportion of tertiary industry to GDP (B20), and water-saving irrigation area (B25) on water ecological security were gradually weakening. This indicates that promoting water-saving irrigation, emphasizing ecological water consumption, and developing the tertiary industry in the Hexi region are crucial for maintaining water ecological security.

**Table 7:** Main barrier factors of water ecological security index layer in the Hexi Corridor.

Obstacle sorting	2006		2007		2008		2008	2009	
	Obstacle factor	Obstacle%	Obstacle factor	Obstacle%	Obstacle factor		Obstacle%	Obstacle factor	Obstacle%
1	B6	8.32	B6	10.18	B10	12.26		B10	12.13
2	B21	7.63	B20	8.43	B6	8.82		B6	9.47
3	B20	7.52	B10	8.29	B20	7.28		B20	7.61
4	B25	6.87	B21	7.68	B21	6.77		B21	7.02
Obstacle sorting	2010		2011		2012			2013	
	Obstacle factor	Obstacle%	Obstacle factor	Obstacle%	Obstacle factor	Obstacle%		Obstacle factor	Obstacle%
1	B10	13.11	B10	11.16	B10	13.14		B10	12.67
2	B6	10.10	B6	9.54	B6	9.78		B6	10.45
3	B20	8.44	B20	8.22	B20	8.46		B20	7.35
4	B16	6.90	B21	7.32	B21	7.26		B25	6.16
Obstacle sorting	2014		2015		2016			2017	
	Obstacle factor	Obstacle%	Obstacle factor	Obstacle%	Obstacle factor	Obstacle%		Obstacle factor	Obstacle%
1	B10	12.59	B10	13.15	B10	14.68		B10	15.63
2	B6	10.65	B6	9.60	B6	9.13		B6	10.08
3	B21	6.64	B21	6.12	B21	6.89		B21	7.38
4	B20	6.57	B14	5.57	B1	5.02		B1	5.55
Obstacle sorting	2018		2019		2020			2021	
	Obstacle factor	Obstacle%	Obstacle factor	Obstacle%	Obstacle factor	Obstacle%		Obstacle factor	Obstacle%
1	B10	18.53	B10	21.20	B10	18.50		B10	11.92
2	B6	8.52	B6	12.72	B15	6.97		B8	9.67
3	B8	6.66	B8	6.55	B8	6.24		B15	7.79
4	B1	5.88	B1	6.51	B1	6.10		B20	7.49

### Prediction of water ecological security in the Hexi Corridor

**Figure 9:**
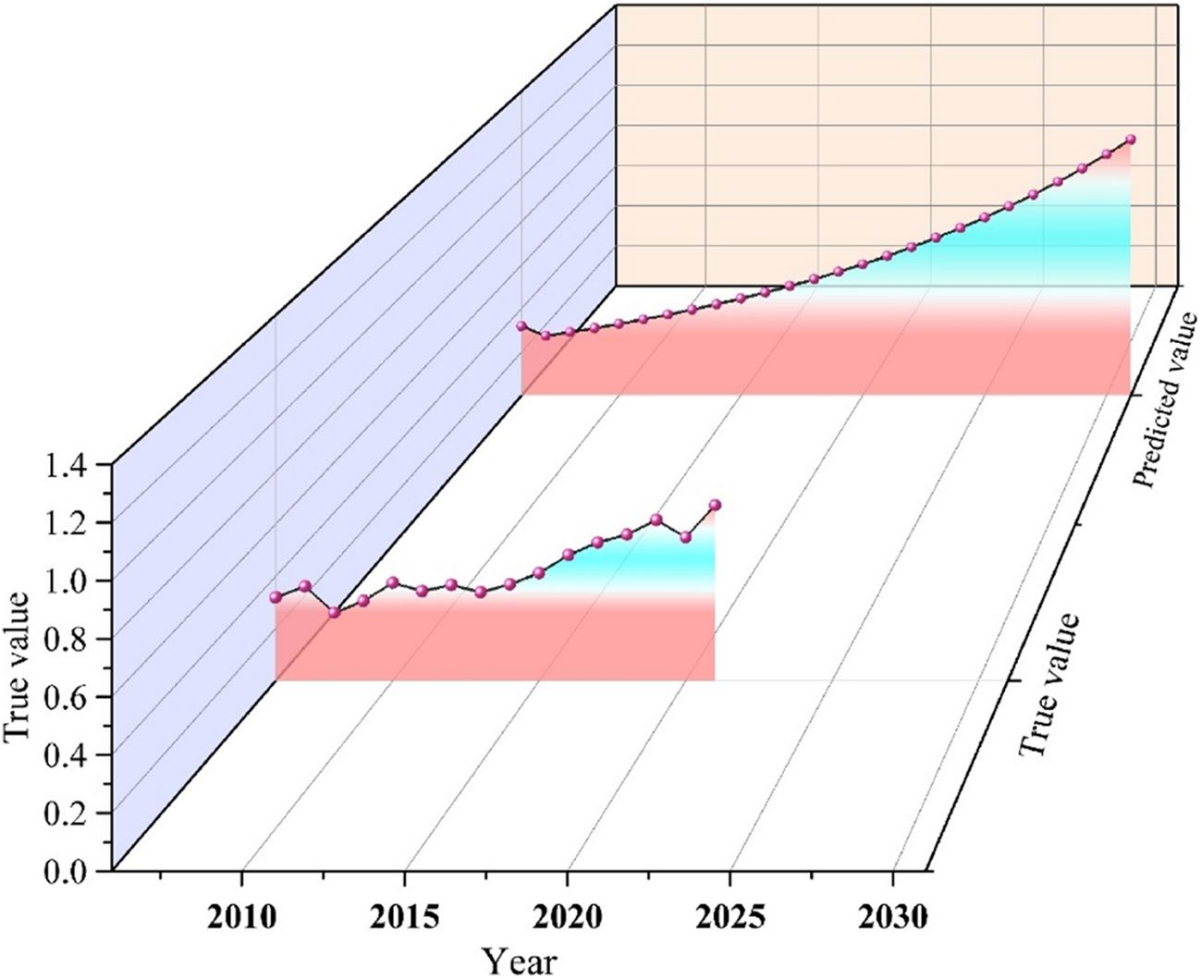
2006-2031 prediction trend of comprehensive index of water ecological security.

Using the GM (1,1) model, we predicted the comprehensive index of water ecological security in the Hexi Corridor from 2022 to 2031. The simulation results had a posterior error ratio of 0.33 and a small error probability of 1.0, both falling within the “good” range for model accuracy. The fitting results were normal, and the simulation closely aligned with the actual data. The fit between the simulated and actual water safety indices was good, demonstrating that the GM (1,1) model is suitable for predicting and analyzing water ecological security in the Hexi Corridor. The prediction results are presented in Table 8; Fig. 9. From 2022 to 2031, water ecological security in the Hexi Corridor is expected to show a continuous upward trend. By 2025, the security level is predicted to improve from relatively safe to safe. The predicted index curve for the Hexi Corridor aligns closely with the actual water ecological security index from 2006 to 2021. From 2022 to 2031, the water ecological security status in the Hexi Corridor is projected to exhibit a steady upward trend, with an average annual growth rate of 4.96%. In the coming years, the PSR system in the Hexi Corridor is expected to develop in a coordinated manner, leading to a steady improvement in water ecological security. The water ecological situation in the Hexi Corridor is expected to improve significantly, with considerable potential for the water ecological security index to increase. However, unpredictable challenges may arise due to force majeure factors such as natural disasters and government actions.

**Table 8:** GM (1,1) Model Water Ecological Security Comprehensive Index Prediction and Authenticity Test.

Year	True value	Predictive value	Year	True value	Predictive value
2006	0.3185		2019	0.6153	0.5681
2007	0.3609		2020	0.5490	0.6037
2008	0.2599		2021	0.6718	0.6415
2009	0.3054		2022		0.6817
2010	0.3745		2023		0.7244
2011	0.3427	0.3493	2024		0.7698
2012	0.3663	0.3712	2025		0.8180
2013	0.3380	0.3945	2026		0.8693
2014	0.3689	0.4192	2027		0.9238
2015	0.4121	0.4455	2028		0.9817
2016	0.4818	0.4734	2029		1.0432
2017	0.5292	0.5030	2030		1.1086
2018	0.5590	0.5346	2031		1.1780

## Discussion

### Analysis of the impact of different policies on water ecological security

The Hexi Corridor serves as a green barrier against desertification in northwest China, particularly a 300 km ecological barrier around Minqin Oasis in Wuwei City. This barrier slows the shifting sands of the Badain Jaran Desert and prevents the merging of the Badain Jaran and Tengger Deserts. Analyzing the dynamic evolution and driving forces of water ecological security in this region is crucial for accelerating comprehensive water management, enhancing ecological security, and fostering high-quality development and protection of the basin. It also helps balance the relationship between water, sand, greenery, and wealth in Gansu Province, facilitating a gradual move towards high-quality development. Existing literature on ecological security in the Hexi area indicates that the water ecological security index has been increasing year by year, consistent with the findings of this paper. The Hexi Corridor’s water ecological security index increased in 2014, driven by national environmental protection policies under the “Belt and Road” initiative. This improvement was supported by both national and regional policies^28,29^.

Additionally, by using the obstacle factor identification model, this paper offers a novel approach for assessing river basin water ecological security. The paper identified that ecological environmental water consumption, urban water consumption, the proportion of the tertiary industry in GDP, and water-saving irrigation are key factors affecting water ecological security. This suggests that improving ecological water use, such as increasing water supply and efficiency, is crucial. Promoting water-saving irrigation is essential for ensuring the safe and orderly development of water ecology. Developing an ecological water-saving agriculture model that provides both environmental protection and economic benefits will be a key trend in future development. Therefore, it is crucial to establish comprehensive supporting policies and evaluation mechanisms. Relevant laws and regulations should be introduced to bolster ecological security measures, establish a stable ecological security framework, and ensure its sustainability. Investments in environmental protection should be increased, with policies supporting the conversion of farmland to forests and grasslands and continued afforestation. Additionally, industrial emission controls should be strengthened, regional advantages utilized, and the regional tertiary industry promoted^30^. In the coming years, as the concept of ecological civilization strengthens and the “Belt and Road” strategy advances, the Hexi Corridor will deepen the implementation of coordinated ecological protection and economic policies, leading to continued improvements in regional ecological security.

### Limitations and prospects

Water ecological security assessment is an interdisciplinary field characterized by ongoing changes, requiring continuous optimization and updates. Constructing the index system should consider the internal dynamics of the composite ecosystem and analyze the comprehensive impacts of economic, social, and natural factors. As the concepts of ecological and water ecological civilization advance, a new direction will be to examine water ecological security from the perspective of the economic-society-nature complex ecosystem. This paper uses the PSR model to analyze the trend in ecological security of incoming water in the Hexi Corridor over the past 16 years. The evaluation results are accurate, indicating that the selected index system for assessing water ecological security has good stability, operability, and scientific validity. However, there are certain limitations. First, the evaluation results are constrained by the limited index data and the relatively short time series. Although it captures evolving trends in ecological security, extending the time series and incorporating independent indicators would yield more actionable policy insights. Second, indicators and safety levels in comprehensive evaluations can be influenced by subjective factors. Future studies should develop water ecological security models tailored to actual conditions, incorporating a broad range of factors including economic, social, resource, and environmental aspects to enhance objectivity and reliability. Third, the evaluation index system for water ecological security must evolve with time. Introducing indicators such as ecosystem health, network advantage, and biological integrity, as well as incorporating diverse biological indicators and evaluation methods, can enhance the assessment of the water ecological environment^31,32^. The evaluation process provides a macro-level direction but lacks a universally applicable rating system for different regions. As science and technology advance, there is significant potential for improving and optimizing the evaluation index system to enhance both accuracy and simplicity.

## Conclusion

This article established an evaluation index system to analyze the weight and obstacle levels of water ecological security indicators in Gansu Province’s Hexi region from 2006 to 2021. It assessed water ecological security over the past 16 years and predicted future trends for the next 10 years, leading to the following conclusions:

(1) Across the system, the state system was the dominant factor for water ecological security in JYG City, with a weight of 40.5%. The remaining systems were all pressure systems, with weights of 50.7% for JQ, 46.3% for ZY, 41.9% for JC, and 42.5% for WW. Excluding 2006, the pressure system consistently had the highest obstacle degree, peaking at 63.24% in 2019. The major obstacle factors throughout the time series were ecological environmental water use and urban environmental water use, while water use per 10,000 yuan of GDP and industrial added value water use per 10,000 yuan were the smallest obstacle factors, mainly due to geographical constraints.

(2) The overall water ecological security in the Hexi Corridor region demonstrated an upward trend. The water ecological security values for the five prefecture-level cities—WW, ZY, JC, JYG, and JQ—showed improvements with some fluctuations. The indices ranged from 0.25 to 0.67 for the Hexi region, 0.22 to 0.69 for WW, 0.23 to 0.73 for JC, 0.22 to 0.69 for ZY, 0.27 to 0.64 for JYG, and 0.28 to 0.59 for JQ. JC City experienced the most significant increase, with a range of 0.50, while JQ City saw the smallest increase, with a range of 0.31. Overall, the eastern part of the region exhibited slightly higher levels than the west, and the evaluation results aligned with the urban functions of each city.

(3) From 2022 to 2031, water ecological security in the Hexi region is projected to show a steady upward trend, with an average annual growth rate of 4.96%. By 2025, the security level is expected to reach a very safe status. The water ecological security index has substantial potential for further improvement, with anticipated coordinated development of the PSR system and enhancements in ecosystem structure.

(4)The evaluation results are constrained by the limited index data and the relatively short time series. The indicators and safety levels in comprehensive evaluations can be influenced by subjective factors. The evaluation process provides a macro-level direction but lacks a universally applicable rating system for different regions. However, in the future research, the system index can be further refined. At the same time, due to the limited access to data, the follow-up study further carried out microscopic analysis from the county scale, considered the five urban areas of Hexi Corridor nested three river basins for safety assessment, and carried out a more systematic and scientific discussion with the help of big data.

In summary, from 2006 to 2021, water ecological security in the Hexi region of Gansu Province has primarily been constrained by regional pressure systems. Its development has shown gradual, slow improvement and is expected to continue along this trajectory. This research provides a basis for sustaining the ecological security of the Hexi oasis system and enhancing the provision of key ecosystem services.

## Data Availability

The datasets used and/or analysed during the current study available from the corresponding author on reasonable request. At the same time, the raw data can be downloaded from the network connections of the Gansu Provincial Bureau of Statistics and the Gansu Provincial Water Resources Department.
